# Does the risk of neurodevelopment disorders in children differ with different ART treatments? A systematic review and meta-analysis

**DOI:** 10.12669/pjms.41.8.12282

**Published:** 2025-08

**Authors:** Hui Li, Fengfeng Qi, Dongchan Chen

**Affiliations:** 1 Hui Li Department of Child healthcare, Huzhou Maternity & Child Health Care Hospital, Huzhou, Zhejiang Province 313000, P.R. China; 2 Feng feng Qi Department of Child healthcare, Huzhou Maternity & Child Health Care Hospital, Huzhou, Zhejiang Province 313000, P.R. China; 3 Dongchan Chen Department of Pediatric, Huzhou Maternity & Child Health Care Hospital, Huzhou, Zhejiang Province 313000, P.R. China

**Keywords:** Assisted reproductive technology, In vitro fertilization, Neurodevelopmental disorders, Autism spectrum disorder, Intellectual disability

## Abstract

**Objective:**

This meta-analysis aimed to investigate the risk of neurodevelopmental disorders in children conceived through assisted reproductive technology (ART), focusing on comparisons of neurodevelopment outcomes in children conceived using intracytoplasmic sperm injection (ICSI) and conventional in vitro fertilization (IVF), as well as using frozen and fresh embryo transfers.

**Methods:**

A systematic literature search of PubMed, Web of Science, Scopus and Embase databases was done for studies focusing on singleton pregnancies, published in the last two decades (from year 2004 onwards to 31st May 2024) and reporting confounder-adjusted effect sizes. Pooled effect sizes were expressed as relative risk (RR) with 95% confidence intervals (CIs). Egger’s test and funnel plots were used to assess publication bias. The certainty of the pooled evidence was assessed using the GRADE approach.

**Results:**

Analysis of the 13 included studies showed that ICSI correlated with a higher incidence of autism spectrum disorder (ASD) in offspring compared to IVF ART (RR 1.36, 95% CI: 1.05, 1.75). However, the risks of attention-deficit/hyperactivity disorder (ADHD), intellectual disability, cerebral palsy and “any” developmental disorder were similar between the groups. There was no significant difference in neurodevelopmental outcomes, such as ASD, intellectual disability and cerebral palsy, in children conceived after frozen or fresh embryo transfer. The overall quality of evidence for these outcomes was judged to be “Low” according to the GRADE assessment criteria.

**Conclusion:**

Based on the low-quality evidence, children conceived through ICSI may be at higher risk of ASD compared to children conceived through IVF, while risks of other neurodevelopmental disorders appear similar. Frozen embryo transfer does not seem to increase the risk of neurodevelopmental disorders in offspring compared to fresh transfer.

***Registration:*** PROSPERO: CRD42024557480).

## INTRODUCTION

Assisted reproductive technology (ART), such as in vitro fertilization (IVF), intracytoplasmic sperm injection (ICSI) and embryo cryopreservation, has become an integral part of infertility treatment.^1^-^3^ As ART procedures have become more refined and widespread, the focus of research has expanded beyond immediate pregnancy outcomes to include the long-term health and developmental outcomes of children conceived through these methods.^4^,^5^ However, while numerous studies focus on the ART-associated risks of multiple pregnancies, preterm birth and congenital anomalies, the potential impact of these technologies on neurodevelopmental outcomes in offspring remains less clear.^6^-^8^ Neurodevelopmental disorders, including cerebral palsy, intellectual impairment, autism spectrum disorders (ASD), attention deficit hyperactivity disorder (ADHD) and behavioral problems, can significantly affect the quality of life and long-term prognosis of affected children. Given the complexity and multifactorial nature of these disorders, it is crucial to understand whether different ART treatments contribute to varying risks.

Outcomes in children born through ART may be affected by several factors, including the specific technique used, the conditions of embryo culture and the stage at which embryos are transferred or cryopreserved.^9^-^11^ However, the research of possible links between various AST procedures and neurodevelopmental outcomes is complicated by the challenges in conducting long-term follow-up studies and delayed manifestation of some neurodevelopmental disorders. Several reviews attempted to summarize known data on these associations.^12^-^15^ A review conducted by Djuwantono et al. (2020) included six studies comparing ICSI with conventional IVF and frozen with fresh embryo transfer^12^ and revealed that children born through ICSI had a higher risk of intellectual disability and ASD compared to children born through conventional IVF.

However, the review did not find significant impact of embryo cryopreservation on the risk of neurodevelopmental disorders.^12^ In another review by Catford et al. (2017), twenty-four studies published between 1995 and 2016 were included, focusing on neurodevelopmental outcomes in children conceived through ICSI compared to IVF.^13^ However, no formal meta-analysis was conducted and findings were presented narratively. While studies suggested comparable neurodevelopment in children, conceived by IVF and ICSI, ICSI was associated with an increased risk of autism and intellectual impairment.^13^ This review did not include a comparison between frozen and fresh embryo transfers.

Importantly, current available synthesized evidence is presented mostly in form of narrative reviews and utilizes findings from studies, irrespective of whether they were adjusted for potential confounders or not. Given heterogeneous and sometimes contradictory findings of available studies, there is a need for a comprehensive updated review to draw more definitive conclusions. The current meta-analysis aimed to systematically evaluate the existing literature on neurodevelopmental outcomes in children conceived through different ART procedures and to compare the risks associated with ICSI versus conventional IVF and frozen versus fresh embryo transfer.

## METHODS

### Search strategy:

A systematic search of PubMed, Web of Science, Scopus and Embase was done to identify studies published until 31st May 2024. Search keywords used were: (reproductive techniques, assisted[mh] OR assisted reproduc*[tw] OR in vitro fertilization[tw] OR in vitro fertilization[tw] OR Intracytoplasmic Sperm Injection (tw) OR frozen embryo transfer [tw] OR frozen embryo transfer[mh] OR fresh embryo transfer[tw] OR fresh embryo transfer[mh]) AND (Child development[mh] OR Child development[tw] OR Cognition[mh] OR Cognition[tw] OR Cognition disorders[mh] OR Developmental disabilities[mh] OR Developmental disabilit*[tw] OR Language disorders[mh] OR Language disorder*[tw] OR Learning disorders[mh] OR Learning disorder* [tw] OR Intelligence[mh] OR Intelligence[tw] OR Intelligence tests[mh] OR child behav*[tw] OR neurobehavioral manifestations[tw] OR neurobehavioral manifestation*[tw]). The search strategy was customized as per the needs of each of the databases. The PRISMA guidelines were followed. Study protocol was pre-registered in PROSPERO; (https://www.crd.york.ac.uk/prospero/),^16^ (reference number: CRD42024557480).

### Screening and selection of the studies:

The initial pool of studies, identified by the search across databases, was deduplicated. Subsequently, unique studies underwent screening of titles and abstracts for relevance by two reviewers (HL & FQ). Full texts of relevant reports were read and eligible studies were included in the final review. To ensure the reliability and accuracy of this selection process, each step was conducted independently by the two authors. Any differences were resolved by consultation with third reviewer (DC).

### Inclusion Criteria:

Original research articles that investigated neurodevelopmental outcomes in children conceived through ART and studies focusing on singleton pregnancies were included. The research had to compare either IVF and ICSI techniques or fresh versus frozen embryo transfers. We included studies published in the last two decades (from 2004 onwards) to ensure that the evidence reflected recent advancements in ART, including improvements in scientific rigor, standardized protocols and technological development.^17^ Additionally, the studies needed to report on outcomes of interest, i.e., cerebral palsy, intellectual impairment, ASD and behavioural problems. Only studies that reported confounder-adjusted effect sizes were included, to minimize bias and ensure the reliability of associations. We also included studies that reported outcomes as mean differences in neurodevelopmental scores between groups, provided these were confounder-adjusted. The studies had to be published in peer-reviewed journals and have a follow-up period extending into early childhood or beyond. There were no language restrictions, although we found no relevant non-English language papers.

### Exclusion Criteria:

Review articles, meta-analyses, case reports, editorials, or conference abstracts were excluded. Studies that did not differentiate between various ART techniques or lacked comparative data on neurodevelopmental outcomes were also excluded. Research focusing exclusively on non-neurodevelopmental outcomes, such as immediate birth outcomes or physical growth metrics, was not considered.

### Data extraction, quality assessment:

Data were extracted independently by the two authors using a structured extraction form, developed collaboratively after thorough discussions. This form captured critical details such as study identifiers (author and year of publication), study design, child’s age at assessment, methods of assessment and diagnosis, adjusted confounders, sample size and key findings. Any disagreements or discrepancies encountered during the extraction process were resolved through consensus discussions, ensuring consistency and accuracy in the collected data. Newcastle-Ottawa Scale (NOS), with a maximum attainable score of 9, was employed to assess study quality.^18^ Studies with scores of ≤6, 7-8 and 9 were considered to be low, moderate and high quality respectively.

### Statistical analysis:

STATA version 15.0. was used for analyses. The pooled effect sizes were reported as relative risk (RR) with 95% confidence intervals (CIs). A random-effects model was used for the analysis to account for potential variations in participant characteristics and methodological differences across the included studies.^19^ Funnel plots and Egger’s test^20^ were used to assess publication biases. Heterogeneity was assessed using I^2^ statistic with values >50% indicating high heterogeneity. P<0.05 was considered significant. We evaluated the certainty of the evidence using the standard GRADE approach and GRADE Pro software.^21^

## RESULTS

**Fig.1:**
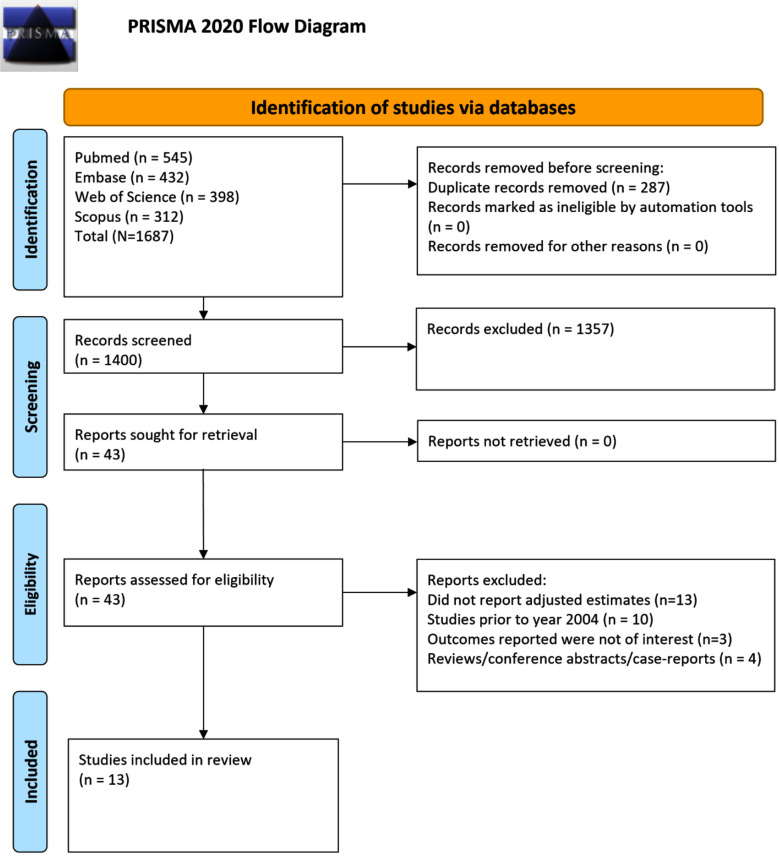
PRISMA flowchart to show process of study selection.

The database search yielded 1687 papers. Of them, 287 duplicate records were removed. Screening of titles and abstracts (Fig.1) eliminated an additional 1357 studies. The full texts of the remaining 43 studies were then reviewed. Ultimately, 13 eligible studies were included in the final meta-analysis (Fig.1).^22^-^34^ The important characteristics of the studies have been presented in Table-I. Most studies were retrospective cohort in design (n=10) and the remaining three reports were matched case-control studies. Three studies were from the USA, three from Netherlands and two from Denmark. One study each was from Canada, Taiwan, Australia and Sweden. One study was multicentric. Most studies reported on neurodevelopment outcomes based on the medical records/ICD-based coding (n=8). The remaining five studies used objective assessment or caregiver’s report (Table-I). Studies included a total of 91,589 children conceived by ICSI and 131,746 children conceived by IVF; 32,173 children conceived after frozen and 155,692 after fresh embryo transfer. All studies were of good quality (mean NOS score of 7.31), with seven studies having an NOS score of 8, three studies having a score of 7 and three studies having a score of 6 (Table-II).

**Table-I:** Summary of included studies.

Author	Study design; location	Comparison between ART techniques	Sample size	Age at assessment	Adjusted for	Diagnosis based on
Velez et al (2023)	RC; Canada	ICSI vs. IVF	ICSI: 2551 IVF: 20968	Mean 3.9 years	Maternal age, parity, income quintile, rurality, immigration status, smoking, obesity, any drug or alcohol use, maternal history of mental illness or ASD, pre-pregnancy diabetes mellitus or chronic hypertension and infant sex	2 or more outpatient diagnoses, by either a pediatrician or psychiatrist and/or 1 or more diagnoses during a hospitalization
Lo et al (2022)	RC; Taiwan	ICSI vs. IVF	ICSI: 3825 IVF: 3889	Mean 5.8 years	Maternal age, paternal age, gestation status (full-term or preterm) and offspring sex	ICD-9 or 10
Rönö et al (2022)	RC; Multicentric (Denmark, Finland, Norway and Sweden)	ICSI vs. IVF Frozen vs. Fresh embryo transfer	ICSI: 37,504 IVF: 52,160	Mean 7.8 years	Offspring sex, mother’s age at delivery, parity, smoking during pregnancy and maternal psychiatric morbidity.	ICD-10
Diop et al (2019)	RC; USA	ICSI vs. IVF	ICSI: 3904 IVF: 4834	0 to 3 years	Maternal age, race, education, marital status, nativity), insurance, smoking, prenatal care, parity, gender, method of delivery, chronic and pregnancy hypertension, gestational and chronic diabetes, breech	ICD-9
Hansen et al (2018)	RC; Australia	ICSI vs. IVF Frozen vs. Fresh embryo transfer	ICSI: 618 IVF: 1291	Median 12.6 years	Offspring sex, year of birth group, parity group, maternal age group, delivery mode and private health insurance	Record based
Kissin et al (2015)	RC; USA	ICSI vs. IVF Frozen vs. Fresh embryo transfer	ICSI: 27901 IVF: 13753	Up to 5 years of age	Infant sex, gestational age, birthweight, maternal and paternal age at delivery, number of previous births, mode of delivery and birth year	Record based (diagnosis based on DSM-IV)
Sandin et al (2013)	RC; Sweden	ICSI vs. IVF Frozen vs. Fresh embryo transfer	ICSI: 9241 IVF: 19445	Mean 10 years	Offspring sex, paternal age, maternal age, maternal psychiatric history at offspring birth, paternal psychiatric history at offspring birth	ICD-9/10
Pinborg et al (2010)	RC; Denmark	ICSI vs. IVF Frozen vs. Fresh embryo transfer	ICSI: 3649 IVF: 7564	Mean 5.4 years	Maternal age, parity, child year of birth and child gender	Hospital records
Zhu et al (2009)	RC; Denmark	ICSI vs. IVF	ICSI: 309 IVF: 1153	Mean 19.5 months	Maternal age, parity, parental occupational status and child’s age	Caregiver reported responses
Knoester et al (2008)	CC; Netherlands	ICSI vs. IVF	ICSI: 83 IVF: 83	5 to 8 years	Paternal education, pregnancy complications	Assessment of the child using Revised Amsterdam Child Intelligence Test (RAKIT)
Knoester et al (2007)	CC; Netherlands	ICSI vs. IVF	ICSI: 81 IVF: 81	5 to 8 years	Paternal smoking during pregnancy, paternal educational level and socio-economic status	Assessment using Child Behaviour Checklist
Knoester et al (2007a)	CC; Netherlands	ICSI vs. IVF	ICSI: 81 IVF: 81	5 to 8 years	Maternal age, parity and low birthweight	Standardized neuromotor examination
Hvidtjørn et al (2006)	RC; Denmark	ICSI vs. IVF Frozen vs. Fresh embryo transfer	ICSI: 1842 IVF: 6444	1 to 7 years of age	Maternal educational level, age, parity, gender, multiplicity and preterm delivery.	ICD-10

ICSI: intracytoplasmic sperm injection; IVF: in vitro fertilization; RC: retrospective cohort; CC: case-control; ICD- International Classification of Diseases.

### Neurodevelopmental outcomes in children conceived by ICSI compared to IVF:

**Fig.2:**
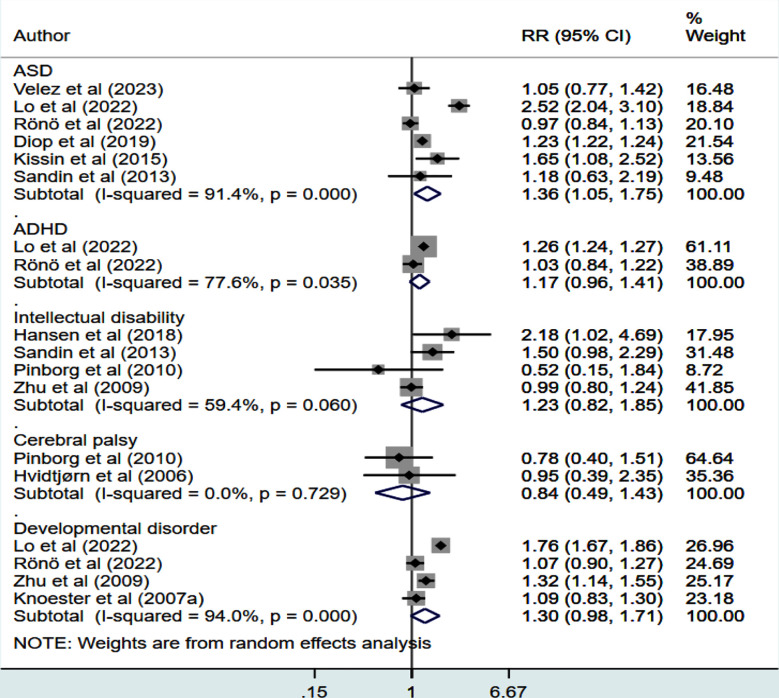
Risk of neurodevelopmental disorders in children born from ICSI compared to conventional IVF.

Compared to offspring conceived through IVF, ICSI conception was associated with the increased risk of developing ASD (RR 1.36, 95% CI: 1.05, 1.75; n=6, I^2^=91.4%) (Fig.2).

The risk of ADHD (RR 1.17, 95% CI: 0.96, 1.41; n=2, I^2^=77.6%), intellectual disability (RR 1.23, 95% CI: 0.82, 1.85; n=4, I^2^=59.4%), cerebral palsy (RR 0.84, 95% CI: 0.49, 1.43; n=2, I^2^=0.0%) and “any” developmental disorder (RR 1.30, 95% CI: 0.98, 1.71; n=4, I^2^=94.0%) was similar between the two groups of children (Fig.2). Publication bias assessment by the Egger’s test and funnel plot was done only for ASD, intellectual disability and “any” developmental disorder because of the small number of studies reporting most outcomes. The Egger’s p-value was statistically non-significant for ASD (p=0.67) and intellectual disability (p=0.68) but significant (p=0.02) for “any” developmental disorder. The overall quality of evidence for the above outcomes was judged to be “Low” according to the GRADE assessment criteria (Table-II).

**Table II:** Risk of bias analysis

Author	Selection of cohort	Comparability	Outcome assessment	Quality score
Velez et al (2023)	4	2	2	8
Lo et al (2022)	4	2	2	8
Rönö et al (2022)	4	2	2	8
Diop et al (2019)	4	2	2	8
Hansen et al (2018)	4	-	2	6
Kissin et al (2015)	4	2	2	8
Sandin et al (2013)	4	2	2	8
Pinborg et al (2010)	4	2	1	7
Zhu et al (2009)	4	2	2	8
Knoester et al (2008)	4	2	1	7
Knoester et al (2007)	4	-	2	6
Knoester et al (2007a)	4	-	2	6
Hvidtjørn et al (2006)	4	2	1	7

### Frozen compared to fresh embryo transfer:

**Fig.3:**
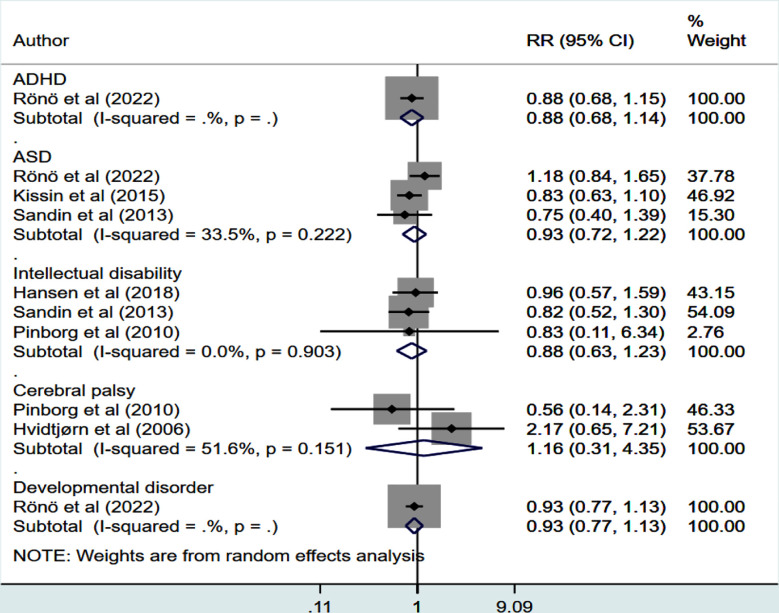
Risk of neurodevelopmental disorders in children born from frozen compared to fresh embryo transfer.

Frozen and fresh embryo transfer were linked to comparable rates of ASD (RR 0.93, 95% CI: 0.72, 1.22; n=3, I^2^=33.5%), intellectual disability (RR 0.88, 95% CI: 0.63, 1.23; n=3, I^2^=0.0%) and cerebral palsy (RR 1.16, 95% CI: 0.31, 4.35; n=2, I^2^=51.6%) in offspring (Fig.3). The overall quality of evidence was judged to be “Low” according to the GRADE assessment criteria (Table-III).

**Table-III:** Assessing the certainty of the evidence using GRADE approach.

Outcomes	Number of studies (study design)	Certainty of the evidence (GRADE)	Relative effect (RR) (95% CI)
***ICSI compared to IVF***			
Risk of ASD	7 (All cohort)	⨁⨁◯◯Low^a^	RR 1.36 (1.05, 1.75)
Risk of ADHD	2 (All cohort)	⨁⨁◯◯Low^a^	RR 1.17 (0.96 to 1.41)
Risk of intellectual disability	4 (All cohort)	⨁⨁◯◯Low^a^	RR 1.23 (0.82 to 1.85)
Risk of cerebral palsy	2 (All cohort)	⨁⨁◯◯Low ^b^	RR 0.84 (0.49 to 1.43)
Risk of “any” developmental disability	4 (All cohort)	⨁⨁◯◯Low^a^	RR 1.30 (0.98 to 1.71)
***Frozen compared to fresh embryo transfer***			
Risk of ASD	3 (All cohort)	⨁⨁◯◯Low ^b^	RR 0.93 (0.72, 1.22)
Risk of intellectual disability	3 (All cohort)	⨁⨁◯◯Low ^b^	RR 0.88 (0.63 to 1.23)
Risk of cerebral palsy	2 (All cohort)	⨁⨁◯◯Low^a^	RR 1.16 (0.31 to 4.35)

***Explanations:***

a) Downgraded two levels for: non-randomized studies; serious inconsistency (high heterogeneity)

b) Downgraded two levels for: non-randomized studies; imprecise results (wide confidence intervals).

Due to limited number of studies, publication bias using Egger’s test and funnel plot was done only for ASD and intellectual disability. The Egger’s p-value was statistically non-significant for ASD (p=0.90) and intellectual disability (p=0.99).

## DISCUSSION

This meta-analysis showed that using ICSI to achieve pregnancy led to slightly higher risk of ASD in offspring compared to IVF. However, the risks of ADHD, intellectual disability, cerebral palsy and “any” developmental disorder were similar in the groups. Similarly, frozen and fresh embryo transfers were associated with a comparable incidence rate of neurodevelopmental disorders in offspring. Our results are consistent with previous reports and reviews^12^,^13^ that also showed that while ICSI conception may increase the incidence of ASD, it does not markedly impact the overall risk of other neurodevelopmental disorders compared to conventional IVF.

The observed increased risk of ASD in children conceived through ICSI warrants further investigation into potential underlying mechanisms. One possible explanation could be related to the inherent differences in the patient populations undergoing ICSI versus IVF. ICSI is often used in cases of severe male factor infertility, where sperm quality may be compromised. Therefore, it is plausible that the underlying genetic or epigenetic factors may contribute to the increased risk of ASD in offspring.^35^,^36^ Additionally, the invasive nature of ICSI, involving direct injection of sperm into the egg, might potentially impact embryonic development and increase the risk of neurodevelopmental disorders.^37^ While ASD showed an increased risk with ICSI, the risks of other developmental disorder were similar between ICSI and IVF groups. This suggests that factors contributing to the risk of ASD may be specific to ICSI and may not influence other neurodevelopmental outcomes to the same extent. However, further research is needed to explore these associations comprehensively.

Importantly, our study suggests that the freezing and thawing process involved in frozen embryo transfer does not impact the risk of neurodevelopmental disorders in offspring compared to fresh transfer. This may be due to advances in cryopreservation techniques that minimize potential damage to embryos during freezing and thawing.^38^,^39^ Our results have an important clinical significance. Studies show that frozen embryo transfer lowers the risk of ovarian hyperstimulation syndrome.^40^ Additionally, fertility drugs were shown to negatively affect the lining of the womb and lower implantation rates.^41^-^43^ Therefore, frozen embryo transfer could be more beneficial than fresh embryo transfer in improving pregnancy rates. However, further high-quality research is needed.

### Limitations.

A small number of studies for some outcomes may have led to the limited ability to detect any significant differences and also affected the robustness of our findings. This is reflected in the “Low” quality of evidence judged according to the GRADE assessment criteria Additionally, heterogeneity across studies and potential publication bias should be acknowledged. The included studies varied in their population characteristics, child age at assessment and method(s) of outcome measurement, which may have contributed to heterogeneity. Particularly, in most studies, assessment of neurodevelopmental disorder was based on medical records or ICD-9/10 coding, which in itself, is a key limitation.

### Clinical utility and future research directions:

Medical professionals should acknowledge the possible increased risk of ASD in children conceived through ICSI and provide appropriate counselling to prospective parents. However, it’s important to note that the absolute risk remains relatively low. Moreover, the choice between frozen and fresh embryo transfer does not appear to impact neurodevelopmental outcomes, which is reassuring for couples undergoing ART procedures. Individualized counseling is essential and should consider specific fertility issues of the patients, risk factors and preferences to make informed decisions about the choice of ART technique. Future studies must also focus on understanding the underlying mechanisms behind the observed associations and identifying modifiable risk factors. Prospective longitudinal large-scale studies with longer follow-up periods need to confirm our findings and provide broader insights into neurodevelopmental outcomes. Comparative effectiveness research of different ART techniques and protocols is warranted to identify the safest and most effective approaches while minimizing the risk of neurodevelopmental disorders. Interventional studies aimed at mitigating potential risks associated with ART procedures should be considered, along with multidisciplinary approaches involving clinicians, embryologists, geneticists and neurodevelopmental specialists, to advance our understanding and guide clinical practice effectively.

## CONCLUSION

Low-quality evidence suggests that while ICSI may be linked to an increased risk of ASD in offspring, overall risks of neurodevelopmental disorders appear similar between ICSI and IVF and between frozen and fresh embryo transfers. These findings should be further tested in methodologically robust high-quality studies.

### Authors’ contributions:

**HL:** Literature search, study design and manuscript writing.

**FQ and DC:** Data collection, data analysis, interpretation and Critical Review.

**HL:** Manuscript revision and validation and is responsible for the integrity of the study.

All authors have read and approved the final manuscript.

## References

[1] Graham ME, Jelin A, Hoon AH, Wilms Floet AM, Levey E, Graham EM (2023). Assisted reproductive technology:Short- and long-term outcomes. Dev Med Child Neurol.

[2] Jain M, Singh M (2024). Assisted Reproductive Technology (ART) Techniques. In:StatPearls.

[3] Huang JYJ, Rosenwaks Z (2014). Assisted reproductive techniques. Methods Mol Biol.

[4] Chen X, Zhou P (2025). Impact of assisted reproductive technology on the risk of cerebral palsy:A systematic review and meta-analysis. Pak J Med Sci.

[5] Liu Z, Yu L, Kang X, Zhang Y, Yan J (2025). Association of assisted reproductive technology with adverse maternal outcome:A cohort study. Pak J Med Sci.

[6] Yang M, Fan XB, Wu JN, Wang JM (2018). Association of assisted reproductive technology and multiple pregnancies with the risks of birth defects and stillbirth:A retrospective cohort study. Sci Rep.

[7] Banica AM, Popescu SD, Vladareanu S (2021). Obstetric and Perinatal Complications Associated with Assisted Reproductive Techniques - Review. Maedica (Bucur).

[8] Dimanlig-Cruz S, Corsi DJ, Lanes A, Meng L, Miao Q, Walker M (2023). Perinatal and pediatric outcomes associated with the use of fertility treatment:a population-based retrospective cohort study in Ontario, Canada. BMC Pregnancy Childbirth.

[9] Castillo CM, Harper J, Roberts SA, O'Neill HC, Johnstone ED, Brison DR (2020). The impact of selected embryo culture conditions on ART treatment cycle outcomes:a UK national study. Hum Reprod Open.

[10] Wang X, Mao R, Wang M, Long R, Jin L, Zhu L (2023). The effect of recryopreservation on embryo viability and outcomes of in vitro fertilization:a systematic review and meta-analysis. Fertil Steril.

[11] Belva F, Blockeel C, Keymolen K, Buysse A, Bonduelle M, Verheyen G (2023). Impact of embryo vitrification on children's health, including growth up to two years of age, in comparison with results following a fresh embryo transfer. Fertil Steril.

[12] Djuwantono T, Aviani JK, Permadi W, Achmad TH, Halim D (2020). Risk of neurodevelopmental disorders in children born from different ART treatments:a systematic review and meta-analysis. J Neurodev Disord.

[13] Catford SR, McLachlan RI, O'Bryan MK, Halliday JL (2017). Long-term follow-up of intra-cytoplasmic sperm injection-conceived offspring compared with in vitro fertilization-conceived offspring:a systematic review of health outcomes beyond the neonatal period. Andrology.

[14] Middelburg KJ, Heineman MJ, Bos AF, Hadders-Algra M (2008). Neuromotor, cognitive, language and behavioural outcome in children born following IVF or ICSI-a systematic review. Hum Reprod Update.

[15] Rumbold AR, Moore VM, Whitrow MJ, Oswald TK, Moran LJ, Fernandez RC (2017). The impact of specific fertility treatments on cognitive development in childhood and adolescence:a systematic review. Hum Reprod.

[16] Page MJ, McKenzie JE, Bossuyt PM, Boutron I, Hoffmann TC, Mulrow CD (2021). The PRISMA 2020 statement:an updated guideline for reporting systematic reviews. BMJ.

[17] Kushnir VA, Smith GD, Adashi EY (2022). The Future of IVF:The New Normal in Human Reproduction. Reprod Sci.

[18] Wells G, Shea B, O'Connell D, Peterson J, Welch V, Losos M The Newcastle-Ottawa Scale (NOS) for assessing the quality of nonrandomised studies in meta-analyses.

[19] Higgins J, Thomas J, Chandler J, Cumpston M, Li T, Page M Cochrane Handbook for Systematic Reviews of Interventions.

[20] Egger M, Davey Smith G, Schneider M, Minder C (1997). Bias in meta-analysis detected by a simple, graphical test. BMJ.

[21] McMaster University and Evidence Prime GRADEpro GDT:GRADEpro Guideline Development Tool [Software].

[22] Velez MP, Dayan N, Shellenberger J, Pudwell J, Kapoor D, Vigod SN (2023). Infertility and Risk of Autism Spectrum Disorder in Children. JAMA Netw Open.

[23] Lo H, Weng SF, Tsai EM (2022). Neurodevelopmental Disorders in Offspring Conceived via In Vitro Fertilization vs Intracytoplasmic Sperm Injection. JAMA Netw Open.

[24] Rönö K, Rissanen E, Bergh C, Wennerholm UB, Opdahl S, Romundstad LB (2022). The neurodevelopmental morbidity of children born after assisted reproductive technology:a Nordic register study from the Committee of Nordic Assisted Reproductive Technology and Safety group. Fertil Steril.

[25] Diop H, Cabral H, Gopal D, Cui X, Stern JE, Kotelchuck M (2019). Early Autism Spectrum Disorders in Children Born to Fertile, Subfertile and ART-Treated Women. Matern Child Health J.

[26] Hansen M, Greenop KR, Bourke J, Baynam G, Hart RJ, Leonard H (2018). Intellectual Disability in Children Conceived Using Assisted Reproductive Technology. Pediatrics.

[27] Kissin DM, Zhang Y, Boulet SL, Fountain C, Bearman P, Schieve L (2015). Association of assisted reproductive technology (ART) treatment and parental infertility diagnosis with autism in ART-conceived children. Hum Reprod.

[28] Sandin S, Nygren KG, Iliadou A, Hultman CM, Reichenberg A (2013). Autism and mental retardation among offspring born after in vitro fertilization. JAMA.

[29] Pinborg A, Loft A, Aaris Henningsen AK, Rasmussen S andersen AN (2010). Infant outcome of 957 singletons born after frozen embryo replacement:the Danish National Cohort Study 1995-2006. Fertil Steril.

[30] Zhu JL, Basso O, Obel C, Hvidtjørn D, Olsen J (2009). Infertility, infertility treatment and psychomotor development:the Danish National Birth Cohort. Paediatr Perinat Epidemiol.

[31] Knoester M, Helmerhorst FM, Vandenbroucke JP, Van der Westerlaken LAJ, Walther FJ, Veen S (2008). Cognitive development of singletons born after intracytoplasmic sperm injection compared with in vitro fertilization and natural conception. Fertil Steril.

[32] Knoester M, Helmerhorst FM, Van der Westerlaken L AJ, Walther FJ, Veen S, Leiden Artificial Reproductive Techniques Follow-up Project (L-art-FUP) (2007). Matched follow-up study of 5 8-year-old ICSI singletons:child behaviour, parenting stress and child (health-related) quality of life. Hum Reprod.

[33] Knoester M, Vandenbroucke JP, Helmerhorst FM, Van der Westerlaken LAJ, Walther FJ, Veen S (2007). Matched follow-up study of 5-8-year-old ICSI-singletons:comparison of their neuromotor development to IVF and naturally conceived singletons. Hum Reprod.

[34] Hvidtjørn D, Grove J, Schendel DE, Vaeth M, Ernst E, Nielsen LF (2006). Cerebral palsy among children born after in vitro fertilization:the role of preterm delivery--a population-based, cohort study. Pediatrics.

[35] Feinberg JI, Schrott R, Ladd-Acosta C, Newschaffer CJ, Hertz-Picciotto I, Croen LA (2024). Epigenetic changes in sperm are associated with paternal and child quantitative autistic traits in an autism-enriched cohort. Mol Psychiatry.

[36] Feinberg JI, Bakulski KM, Jaffe AE, Tryggvadottir R, Brown SC, Goldman LR (2015). Paternal sperm DNA methylation associated with early signs of autism risk in an autism-enriched cohort. Int J Epidemiol.

[37] Griffiths TA, Murdoch AP, Herbert M (2000). Embryonic development in vitro is compromised by the ICSI procedure. Hum Reprod.

[38] Bosch E, De Vos M, Humaidan P (2020). The Future of Cryopreservation in Assisted Reproductive Technologies. Front Endocrinol (Lausanne).

[39] Vanderzwalmen P, Ectors F, Panagiotidis Y, Schuff M, Murtinger M, Wirleitner B (2020). The Evolution of the Cryopreservation Techniques in Reproductive Medicine-Exploring the Character of the Vitrified State Intra- and Extracellularly to Better Understand Cell Survival after Cryopreservation. Reprod Med.

[40] Shi Y, Sun Y, Hao C, Zhang H, Wei D, Zhang Y (2018). Transfer of Fresh versus Frozen Embryos in Ovulatory Women. N Engl J Med.

[41] Blanco-Breindel MF, Singh M, Kahn J (2024). Endometrial Receptivity. In:StatPearls.

[42] Zaat T, Zagers M, Mol F, Goddijn M, Van Wely M, Mastenbroek S (2021). Fresh versus frozen embryo transfers in assisted reproduction. Cochrane Database Syst Rev.

[43] Wei D, Liu JY, Sun Y, Shi Y, Zhang B, Liu JQ (2019). Frozen versus fresh single blastocyst transfer in ovulatory women:a multicentre, randomised controlled trial. Lancet.

